# Calculation method and evaluation of surrounding rock pressure of vertical shaft

**DOI:** 10.1038/s41598-024-58516-7

**Published:** 2024-04-02

**Authors:** Zhongxi Tian, Yuanwu Sun, Qingshuang Zhao, Boliang Zhang, Wanrong Liu, Xutao Zhang

**Affiliations:** College of Architecture and Civil Engineering, Liaocheng University, Liaocheng, 252000 Shandong China

**Keywords:** Surrounding rock pressure, Vertical shaft, Axisymmetric layering method, Inclined rock strata, Engineering, Civil engineering

## Abstract

The surrounding rock pressure of vertical shafts is one of the basic parameters of shaft lining design. Investigating its calculation methods and applicable scopes has great engineering significance. The paper classifies and compares the calculation methods, discusses the application scopes of various calculation methods, and proposes that the axisymmetric layered method is highly consistent with the field monitoring data for the calculation of surrounding rock pressure of vertical shafts in bedrock sections on the basis of practical engineering examples. On the basis of Terzaghi theory, the calculation formula of surrounding rock pressure of vertical shaft in inclined rock strata with single group joints is derived. The formula can reflect the influence of rock strata dip angle and joints.

## Introduction

The shaft lining is the hub of underground construction space and ground connection. The stability of shaft lining plays an important role in the construction of urban underground space. The surrounding rock pressure in vertical shaft is one of the basic parameters of shaft lining design. Therefore, analyzing the surrounding rock pressure to optimize the shaft lining structure design and ensure shaft lining and construction operation safety has great significance^1^.

Zhou et al.^2^ simulated the stress state of shaft lining under the bottom drainage condition through a model test and inverted the vertical additional stress distribution along the shaft axis generated by soil. Chou et al.^3,4^ measured the shaft lining stress in the tangential direction of the inner edge on site by embedding strain gauges and then deduced the surrounding rock pressure according to the distribution of the stresses along the shaft lining. Wang et al.^5^ used the stress and deformation of shaft lining in the field test to invert the surrounding rock pressure. Combined with the motion modes and radial deflection of the circular diaphragm wall during excavation, Zhang et al.^6^ introduced a semi-analytical solution to the lateral earth pressure by using three-dimensional horizontal and vertical stress equilibrium for the differential element of the cohesive surrounding soil. By monitoring shaft grouting, Öge İ F^7,8^ verified the prediction of grouting amount by multiple regression modeling and adaptive neural fuzzy reasoning system, and studied the supporting effect and characteristics of radial clearance flexible support system on shaft wall. Yang et al.^9^ studied the bearing characteristics and stability mechanism of macroscopic surrounding rock pressure arch in deep buried thick weakly consolidated strata. Many scholars have conducted in-depth research on the calculation of the soil lateral pressure of vertical shafts in topsoil and deep topsoil, and achieved significant results. However, the research results on the surrounding rock pressure in the bedrock section of vertical shafts are few, and the analysis and evaluation of the applicable scope of theory calculation are limited.

Chen^10^ proposed that the surrounding rock of vertical shafts should be regarded as a viscoelastic body, and the viscoelastic solutions of different models can be obtained through Laplace transformation. Benus^11^ and Board^12^ concluded that the surrounding rock pressure is closely related to the rock strata properties in the actual measurement of the 1800-m horizontal section of the North Idaho shaft lining. Altounyan^13^ concluded that the surrounding rock pressure is very small except for the water pressure in the shaft lining pressure test of the deep-water bearing rock layer of the vertical shaft. Chehadeh, Turan, and Abed^14^ reported that the maximum compressive stresses of the shaft lining increased by a large margin, and significant tensile stress zones emerged when the bedrock inclinations exceeded the 20 dip angle. Through the establishment of a shaft surrounding rock mechanical model, Hu et al.^15^ quantitatively analyzed the displacement of the broken surrounding rock during shaft construction under the coupling action of ground pressure and super-thick crush belt strata. Huang et al.^16^ generated a discrete fracture network using the DFN in FLAC3D6.0, established a dual medium model considering the distribution of large fractures, and studied the safety of the shaft lining under non-uniform pressure. For the calculation of the surrounding rock pressure in inclined strata, few formulas consider the mechanical properties and geometric characteristics of joints.

## Calculation theory of surrounding rock pressure in uniform horizontal vertical shaft

For the geological shaft construction in rock strata with a small dip angle, it is generally treated as an approximate level, without considering the formation tectonic stress, and the surrounding rock pressure is set at a uniform level. In the past, the establishment of empirical formula is often based on some theoretical assumptions or the statistical analysis of certain measured data. The empirical formula based on limit equilibrium and elastic–plastic theories is widely used in vertical shaft confining pressure theory^17^.

### Limit equilibrium theory based on loose medium

Limit equilibrium theory based on loose medium solves the radial pressure according to limit equilibrium conditions, including the active earth pressure theory of plane retaining wall, space axisymmetric limit equilibrium theory, and the arch effect theory.

1. The formula of surrounding pressure of the plane retaining wall is derived from the theory of active earth pressure of the plane retaining wall. The formulas of surrounding pressure, such as Plotogyakolov’s^18^, Zinbarevich’s^19,20^, and suspension theory, belong to this category. The groundwater pressure on the shaft lining pressure is ignored in Pu’s formula, so the formula is unsuitable for the calculating the confining pressure in deep aquifer. Zinbarevich’s formula puts forward a layered calculation based on Pu’s formula, and the influence on groundwater is realized by increasing the value of the lateral pressure coefficient. Suspension theory is evolved from the Zinbarevich’s formula. The pressure of the surrounding is divided into two parts: the earth pressure and soil water pressure of suspended soil. The pressure of the surrounding rock above the groundwater level is still calculated according to Zinbarevich’s formula.
  The formula of heavy liquid adopts the law of static liquid, which takes the water saturated soil layer as a simple heavy liquid to calculate the shaft wall confining pressure. The wall confining pressure calculated by the heavy liquid surrounding rock pressure formula has a linear relationship with the depth. However, because the proportion coefficient is constant, it does not change with the change of formation state, and the calculated value is often greater than the measured value.
2. On the basis of space axisymmetric limit equilibrium and arch effect theories, the theory of a plane retaining wall is unsuitable for the shaft lining and its surrounding soil. The shaft lining is cylindrical, and the soil movement around the shaft lining is a spatial problem rather than a simple plane problem. In the case of shaft lining failure in thick topsoil, the surrounding soil has obvious spatial characteristics, and the shallow topsoil and the deep soil layer slide inward in a cone shape and a cylinder shape, respectively. The stress mechanism of the soil movement around the shaft lining is due to the wedge-shaped effect and the ring effect from squeezing the soil around the shaft lining, that is, the circumferential stress. The existence of the anti-shear force between the unit soil and shaft lining and the surrounding rock and soil forms an arch effect in the vertical direction, preventing the complete transfer of the sliding soil’s weight, and its stress will not increase linearly with the depth. Therefore, the space axisymmetric limit equilibrium theory of wellbore is also called ring effect theory. Berezantsev’s formula^21^ of active earth pressure on cylindrical retaining wall and Ma Yingming’s formula^17^ of earth pressure on sandwich walls are based on this theory.

**Figure 1:**
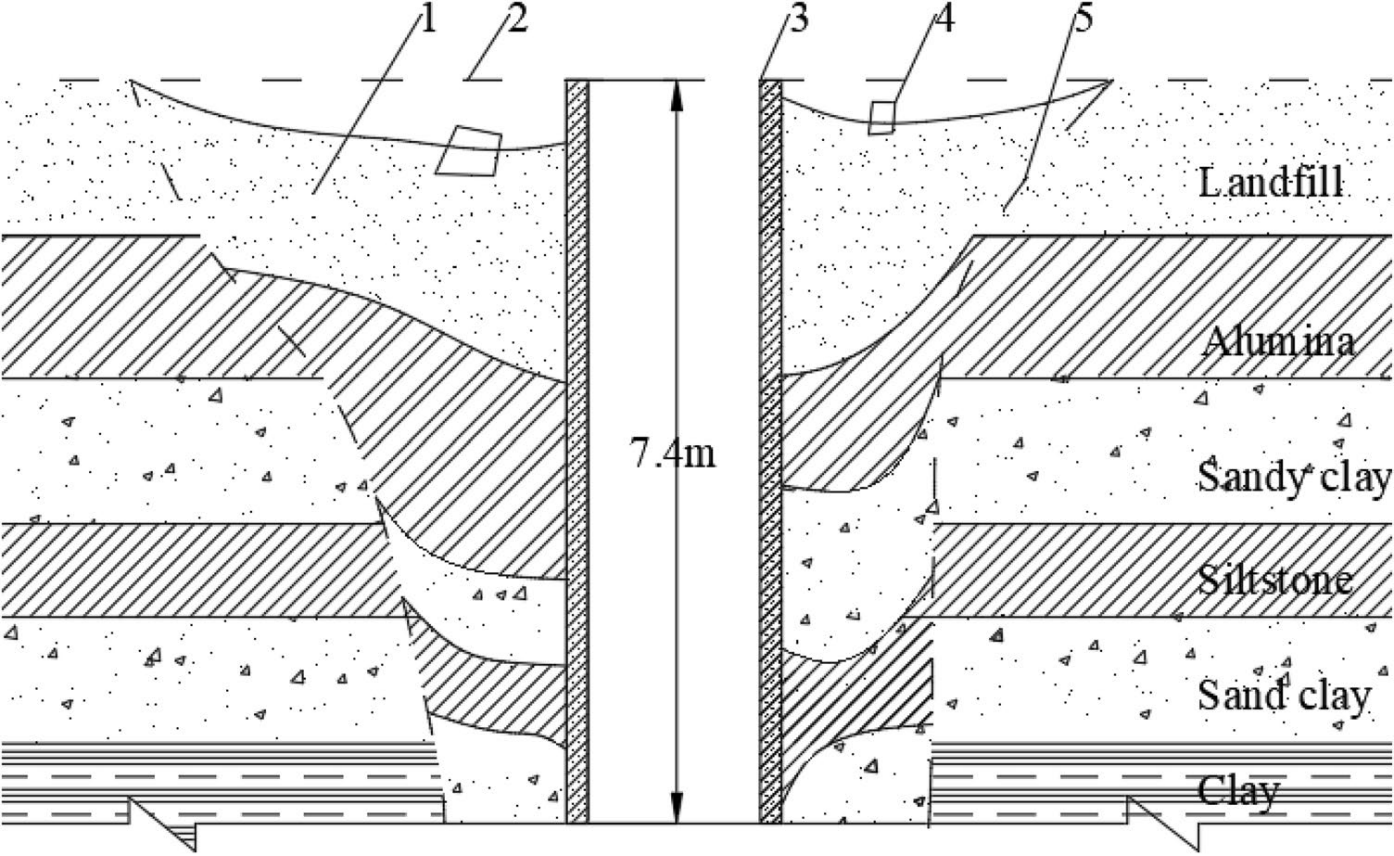
Sliding situation of soil layer near a certain vertical shaft^17^. 1—Area of subsidence, 2—Geological horizon before subsidence, 3—Shaft (clear distance 6.2 m,Wall thinkness 0.6 m), 4—Derrick foundation, 5—Sliding surface outside the area of subsidence.

In the construction of some shafts using the sinking and freezing methods, when collapse accidents occur, the actual exploration of layer displacement settlement is shown in Fig. 1.

**Figure 2:**
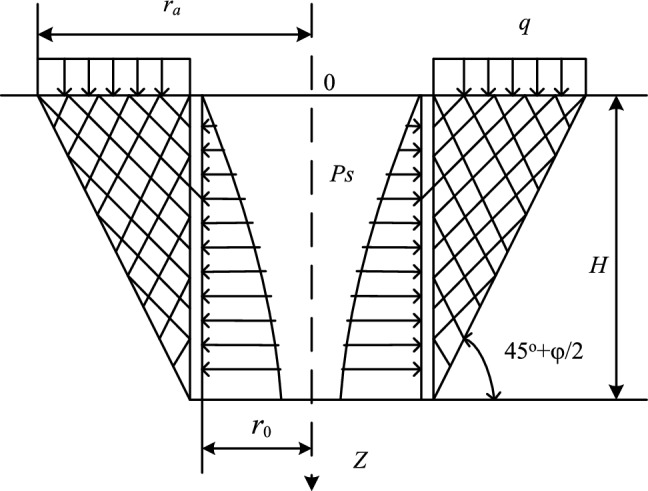
Calculation model of cylinder-shaped retaining wall.

It can be seen that the surface soil layer activity in the shallow part of the shaft is conical, while the activity in the deep part is cylindrical. This is because the soil layer around the shallow shaft is loose, and the shear strength of the soil is small. The soil may slide along the sliding surface at the intersection angle $45^\circ + \frac{\varphi }{2}$ with the horizontal plane, $\varphi$ is the internal friction angle of the soil, and the sliding soil is conical. In the deep, the soil layer is relatively dense, and the shear strength of the soil around the vertical shaft is high. The sliding soil presents a cylindrical shape. Berezantsev considers the vertical shaft and surrounding soil as a spatial structure, treats the sliding soil as a hollow circular cone, and establishes a spatial axisymmetric limit equilibrium calculation model shown in Fig. 2, and established the earth pressure formula of the cylindrical retaining wall combined with the column coordinate system. The formula is as follows:

1 $$\begin{aligned} p_{s} = & \gamma r_{0} \frac{{\tan (45^\circ - \frac{\varphi }{2})}}{{\xi_{0} - 1}}\left[ {1 - (\frac{{r_{0} }}{{r_{a} }})^{{\xi_{0} - 1}} } \right] \\ & + q(\frac{{r_{0} }}{{r_{a} }})^{{\xi_{0} }} \tan^{2} (45^\circ - \frac{\varphi }{2}) \\ & + C{\text{ctan}}\varphi [(\frac{{r_{0} }}{{r_{a} }})^{{\xi_{0} }} \tan^{2} ({45}^\circ - \frac{\varphi }{2}) - {1}], \\ \end{aligned}$$

where, $r_{0}$ is the shaft radius, $q$ is the uniform surface load, $r_{a}$ is the abscissa of the intersection point between the surface and the soil slip line, $\xi_{{0}}$ is the simplified coefficient, that is, $\xi_{0} = 2\tan \varphi \tan (45^\circ + \frac{\varphi }{2})$, $C$ is the cohesion of soil.

**Figure 3:**
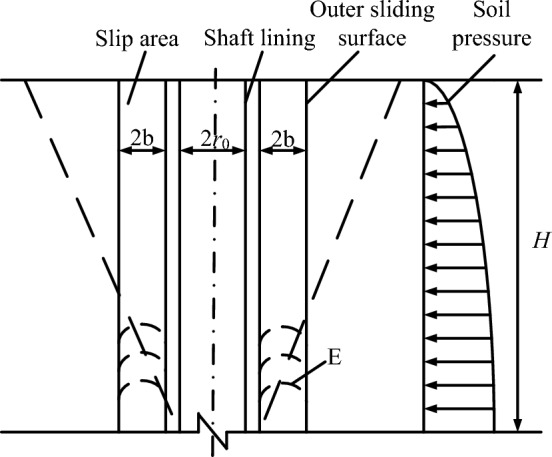
Calculation model of sandwich wall theory.

According to a field investigation and a model test, Ma^2^ proposed sandwich wall theory, which is also called the arch effect theory. For a deep vertical shaft, the change of the stress state leads to the production of a cylindrical sliding body. The lateral pressure of the sliding soil on the shaft lining is the earth pressure of the deep vertical shaft, as shown in Fig. 3.

According to the principle of arch action between two rigid walls in soil mechanics^17^, the formula of arch effect earth pressure (calculation formula of sandwich wall earth pressure) is obtained:

2 $$\sigma_{{\text{h}}} = \frac{{r_{0} \gamma - C}}{tan\varphi }\left( {{1} - e^{{ - \frac{{K_{{\text{a}}} Htan\varphi }}{{r_{0} }}}} } \right),$$

where, $K_{{\text{a}}}$ is the lateral pressure coefficient of the soil body, $K_{{\text{a}}} = tan^{2} ({45}^\circ { - }\frac{\varphi }{2})$.

### Axisymmetric delamination method

**Figure 4:**
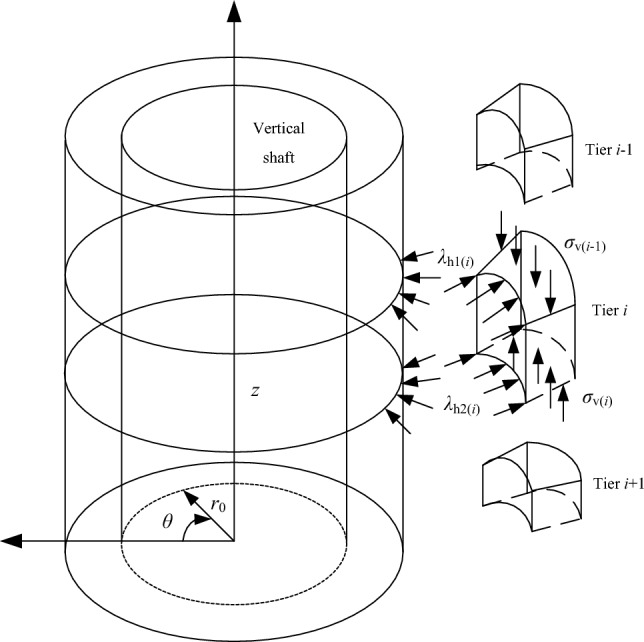
Calculation model of axisymmetric stratification.

Based on the concept of stress transfer in Terzaghi theory, Wang^5,22,23^ proposed an axisymmetric delamination method. The surrounding rock is assumed to be homogeneous and isotropic, and the strength follows the M–C criterion. After excavating the vertical shaft, a fracture zone is formed around the surrounding rock. The fracture radius $R_{{\text{p}}}$ of the vertical shaft is calculated according to the Kastelner equation. The maximum fracture radius $R_{{{\text{pi}}}}$ of each layered rock mass is taken as the fracture radius of the rock strata. The delamination fracture zone is assumed to be a concentric cylinder with different radii, the load of the upper rock column is vertically transferred to the next rock column vertically, and the friction and lateral pressure of the rock column sliding along shaft lining surface are considered. According to Terzaghi theory, the surrounding rock pressure (the lateral pressure of surrounding rock on shaft lining) is calculated when each rock column is in the limit state. The calculation model is shown in Fig. 4.

If the vertical shaft passes through *n* layers of rock mass (the first layer is the topsoil, and it is still calculated according to the Berezantsev formula), the surrounding rock pressure of layer *i* (*i* = 2,3, …, n) in the bed rock is as follows.

The top pressure of layer *i* is

3 $$\sigma_{{{\text{h1(}}i{)}}} = \sigma_{{{\text{v}}(i - 1)}} \times K_{{{\text{a}}i}}{\prime} .$$

The bottom pressure of layer *i* is

4 $$\sigma_{{{\text{h}}2(i)}} = \sigma_{{{\text{v}}(i)}} \times K_{{{\text{a}}i}}{\prime} .$$

In the formulas above, $\sigma_{{{\text{v}}(i)}}$ is the vertical pressure transmitted from the rock column *i* to the rock column* i* + 1;$\sigma_{{{\text{v}}(i - 1)}}$ is the vertical pressure transmitted from the rock column *i*-1 to the rock column *i*. According to the stress state of a single-layer rock mass and the Mohr–Coulomb criteria, the lateral pressure coefficient $K_{{{\text{a}}i}}{\prime}$ of the axisymmetric delamination method is calculated, $K_{{{\text{a}}i}}{\prime} = \frac{1}{{1 + 2\tan^{2} \varphi_{i} }}$, $\varphi_{i}$ is the internal friction angle of layer *i* rock column;

5 $$\begin{gathered} \sigma_{{{\text{v}}(i)}} = \frac{{\gamma_{i} (R_{pi}^{2} - r_{{0}}^{2} )}}{{2R_{pi} \times K_{{{\text{a}}i}}{\prime} \times \tan \varphi_{i} }} - \hfill \\ \left[ {\frac{{\gamma_{i} (R_{pi}^{2} - r_{{0}}^{{2}} )}}{{2R_{pi} \times K_{{{\text{a}}i}}{\prime} \times \tan \varphi_{i} }} - \sigma_{{{\text{v}}(i - 1)}} } \right] \times e^{{ - \frac{{2R_{pi} \times K_{{{\text{a}}i}}{\prime} \times \tan \varphi_{i} }}{{R_{pi}^{i} - r_{{0}}^{{2}} }} \times Z_{1} }} \hfill \\ \end{gathered}$$

When $$Z_{i} \ge \frac{{3(R_{pi}^{2} - r_{0}^{2} )}}{{2R_{pi} \times K_{{{\text{a}}i}}{\prime} \times \tan \varphi_{i} }}$$_,_

6 $$\sigma_{{{\text{v}}(i)}} = \frac{{\gamma_{i} (R_{pi}^{2} - r_{0}^{2} )}}{{2R_{pi} K_{{{\text{a}}i}}{\prime} \tan \varphi_{i} }}.$$

When the original rock lateral pressure coefficient $\lambda = 1$, the radius of the fracture circle $R_{Pi}$ is:

7 $$R_{Pi} = \left[ {\frac{{(p_{0i} + D_{i} {\text{ctan}}\varphi_{i} )(1 - \sin \varphi_{i} )}}{{D_{i} {\text{ctan}}\varphi_{i} }}} \right]^{{\frac{{1 - \sin \varphi_{i} }}{{2\sin \varphi_{i} }}}} \times r_{0} ,$$

where, $P_{01}$ is the The self-weight of the overburden of layer *i* and above, KN/m^2^; $D_{i}$ is the internal cohesion of layer *i* rock mass; and $Z_{i}$ is the thickness of layer *i* rock mass, m.

### Discussion on applicability of calculation methods

The above formulas are used to calculate the surrounding rock pressure of the the vertical shaft in Shiji coal mine in Shandong Province, China. The surrounding rock pressure calculation results are compared with the measured results in the literature^1^, and the application scope of the calculation formula is discussed.

The Shiji coal mine’s vertical shaft passes through the Quaternary topsoil, Jurassic, Carboniferous Permian, and other strata. The topsoil is 7 m in unconformable contact with the lower layer. The Jurassic strata are mainly red sandstone with low strength, poor cementation, and cementation. The Shihezi formation is mainly composed of sandstone, which conform to the Shanxi formation. The Shanxi formation is composed of sandstone and clay, with high joints density and a relatively broken rock mass. The measuring points are set below each layer of rock mass, with six measuring areas and five measuring points in each area. The self weight of the rock mass is taken as the vertical original rock stress, the radial stress of the original rock on the cross section of vertical shaft is assumed to be evenly distributed, and the horizontal lateral pressure coefficient is taken as 0.5.

The calculation results are shown in Table 1.

**Table 1:** Calculation results contrast of pressure.

Rock strata	Depth of measuring point/m	Calculation results of surrounding rock pressure/MPa					
		Heavy liquid formula	Qin's formula	Cylindrical retaining wall theory	Sandwich wall theory	Axisymmetric layered method	Measured values
Siltstone	178	2.3140	1.8650	1.7950	1.6155	0.4395	0.1380
Medium sandstone	221	2.8730	2.176	2.005	1.7243	0.5130	0.2580
Coarse sandstone	280	3.6400	2.690	2.3216	1.9733	0.4890	0.2790
Variegated siltstone	327	4.2510	3.585	2.7204	2.3123	0.9495	0.6375
Medium sandstone	382	4.9660	3.663	3.0109	2.5291	0.8610	0.7815
Fine sandstone	428	5.5640	4.010	3.1182	2.6192	0.8460	0.7935

1. As shown in Fig. 5, based on the theory of the heavy liquid formula, the surrounding rock pressure calculated by the formula is linearly proportional to the depth of the vertical shaft, which is obviously quite different from engineering practice in which the calculated values are larger than the measured values. Therefore, the heavy liquid formula is applicable in the shallow soil layer but not in the thick soil layer and the bedrock section. Guo^24^ introduced automatic monitoring and wireless transmission technology to test the surrounding rock pressure of a hard rock deep vertical shaft. The measured value is 24–46% of the calculated value using Qin’s formula, which is basically consistent with the measured results for the Shiji coal mine.

**Figure 5:**
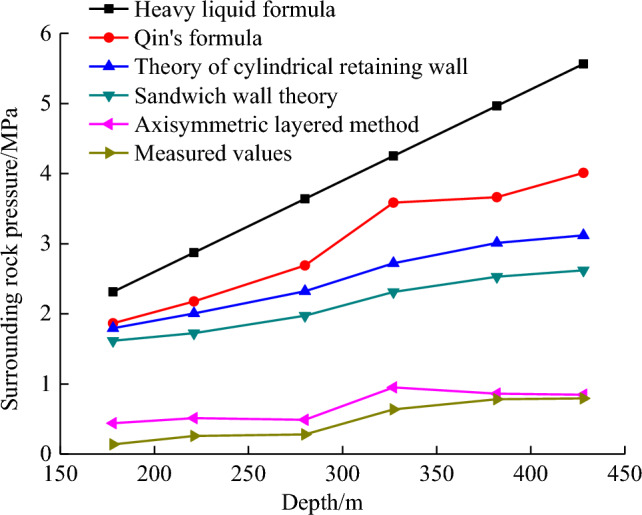
Comparison of ground pressure calculated.
2. The theoretical calculation value of the cylindrical retaining wall has a nonlinear growth relationship with the depth of the shaft lining. The growth rate of the retaining wall decreases gradually with the increase of the depth, and the change trend is consistent with the measured value, but the growth rate is still far greater than the measured value.
3. Based on the pressure cylindrical retaining wall theory, the ground load $q$ is set to 0, and the influence of cohesion $C$ is ignored. The formula is applicable to shallow soil with low cohesion $C$, and the surrounding rock pressure calculation of deep vertical shaft is different from the reality. If the influence of cohesion $C$ is considered, the calculation result of the surrounding rock pressure ($P_{{\text{s}}}$) of the vertical shaft is negative for clay or rock with a large cohesion $C$ because $r_{a}$ exceeds $r_{0}$ with the increase of $H$, indicating that earth pressure cylindrical retaining wall theory is unsuitable for clay and rock with a large cohesion $C$. In addition, when $H \to \infty$, the lateral pressure of soil $\sigma_{{\text{h}}}$ will be equal to a constant, and Eq. (1) is changed to
  8 $$\sigma_{{{\text{h}},\max }} = r_{0} \gamma \frac{{\tan (45^\circ - \frac{\varphi }{2})}}{{K_{{\text{a}}} - 1}}.$$
  When $\varphi \le 19^\circ 30^{\prime}$_,_ $K_{{\text{a}}} \le 1$_,_ and the $\sigma_{{\text{h}}}$ of the vertical shaft is negative, Eq. (1) is no longer applicable.
4. When using sandwich wall theory, cohesion $C$ is often set to 0. If the effect of cohesion $C$ is considered, $r_{0} \gamma - C$ will become negative in cohesive soil or rock mass with a large cohesion $C$, and $\sigma_{{\text{h}}}$ will also be negative, which is inconsistent with reality. For example, if the shaft radius is 1.5 m, the shaft wall thickness is 0.3 m, the soil gravity is 18 kN/m^3^, and cohesion $C$ ≥ 19.44 kPa, then $r_{0} \gamma - C$ is negative. Therefore, sandwich wall theory is inapplicable to clays and rock formations with a large cohesion $C$.
  According to Eq. (2), when $H \to \infty$, the max lateral pressure of soil $\sigma_{{\text{h,max}}}$ will be equal to a constant:
  9 $$\sigma_{{\text{h,max}}} = \frac{{r_{0} \gamma - C}}{\tan \varphi }.$$
  On the basis of Eq. (9), the calculated value of the surrounding rock pressure has a great correlation with wellbore radius $r_{0}$.
5. According to cylindrical retaining and sandwich wall theories, the surrounding rock pressure in the vertical shaft has a power function relationship with the depth in the shallow part and an exponential function relationship in the deep part. With the increase in depth, the growth rate gradually decreases and finally approaches a certain constant. Whether this trend is consistent with reality requires further analysis.
6. For the bedrock section of the vertical shaft, the theory based on loose medium is no longer applicable, and the calculation results of the axisymmetric layered method are near the measured results. The surrounding rock pressure is related to the rock mass properties, but the overall trend increases with the depth, which is consistent with the maximum shear stress and plastic zone proposed near the soft and hard rock interface by numerical simulation^25,26^.

## Calculation of surrounding rock pressure of inclined rock shaft based considering joints

### Surrounding rock pressure calculation of inclined rock circular shaft according to plane problem

**Figure 6:**
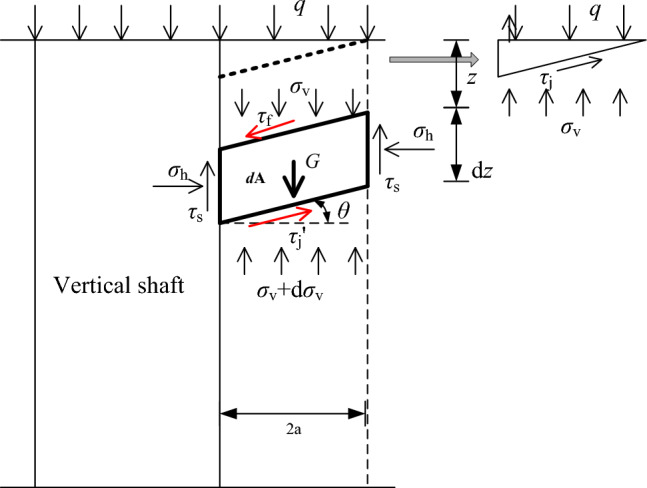
Calculation model of axisymmetric stratification.

Terzaghi theory, which is based on the concept of stress transfer, analyzes the stress state of the micro unit body, calculates the vertical pressure of the rock column, and the surrounding rock pressure (lateral pressure) in the vertical shaft can be obtained from the side pressure coefficient. By using this method, the inclined rock layer is further considered according to the limit equilibrium condition of micro unit. The assumption is the shaft is located in a group of inclined rock strata with the same occurrence, the dip angle of the rock strata is $\theta$, and the friction coefficient of the rock strata is $f = \tan \varphi$. The stress state of the micro unit under the inclined rock condition is analyzed, taking micro unit ${\text{d}}A$ as the research object given that the unit above ${\text{d}}A$ tends to slide downward, ${\text{d}}A$ receives the downward friction stress $\tau_{{\text{f}}}$ from the upper unit and the upward friction stress $\tau_{{\text{f}}} ^{\prime}$ from the lower unit, and $\sigma_{{\text{v}}}$ is the vertical stress of ${\text{d}}A$. The calculation model is shown in Fig. 6.

$\tau_{{\text{f}}}$ can be expressed as

10 $$\tau_{{\text{f}}} = \sigma_{{\text{v}}} f\cos \theta .$$

$\tau_{{\text{f}}} ^{\prime}$ can be expressed as

11 $$\tau_{{\text{f}}} ^{\prime} = (\sigma_{{\text{v}}} + {\text{d}}\sigma_{{\text{v}}} )f\cos \theta .$$

According to stress state of ${\text{d}}A$, the equilibrium differential equation can be established as

12 $$2r_{0} \gamma {\text{d}}z = \frac{{2r_{0} }}{\cos \theta }(\sigma_{{\text{v}}} + d\sigma_{{\text{v}}} ) - \frac{{2r_{0} }}{\cos \theta }\sigma_{{\text{v}}} + 2\tau_{{\text{s}}} {\text{d}}z - 2\sigma_{{\text{v}}} r_{0} f\sin \theta + 2(\sigma_{{\text{v}}} + d\sigma_{{\text{v}}} )r_{0} f\sin \theta ,$$

where, $$\tau_{{\text{s}}} = C + \sigma_{{\text{h}}} \tan \varphi$$ and $$\sigma_{{\text{h}}} = K_{{\text{a}}}{\prime} \sigma_{{\text{v}}}$$, $$K_{{\text{a}}}{\prime} = \frac{1}{{1 + 2\tan^{2} \varphi }}$$.

The boundary conditions are $z = 0,\sigma_{{\text{v}}} = q$, and the surrounding rock pressure of the vertical shaft can be expressed as

13 $$\sigma_{{\text{h}}} = \left[ {K_{{\text{a}}}{\prime} q - \frac{{r_{0} (\gamma - \frac{C}{a})}}{\tan \varphi }} \right]{\text{e}}^{{ - z\frac{{K_{{\text{a}}}{\prime} \tan \varphi }}{{r_{0} }} \times \frac{2\cos \theta }{{2 - f\sin 2\theta }}}} + \frac{{r_{0} (\gamma - \frac{C}{a})}}{\tan \varphi },$$

where, let $k_{{\text{f}}} = \frac{2\cos \theta }{{2 - f\sin 2\theta }}$, and Eq. (13) becomes

14 $$\sigma_{{\text{h}}} = \left[ {K_{{\text{a}}}{\prime} q - \frac{{r_{0} (\gamma - \frac{C}{a})}}{\tan \varphi }} \right]{\text{e}}^{{ - z\frac{{K_{{\text{a}}}{\prime} \tan \varphi }}{{r_{0} }} \times k_{{\text{f}}} }} + \frac{{r_{0} (\gamma - \frac{C}{a})}}{\tan \varphi },$$

$k_{{\text{f}}}$ is the influence coefficient of the rock strata dip angle. When $\theta = 0$, the rock strata dip angle has no influence on the surrounding rock pressure, and Eq. (14) also degenerates to the Terzaghi theoretical calculation formula^27^.

### Calculation of surrounding rock pressure of inclined rock circular shaft according to spatial problems

**Figure 7:**
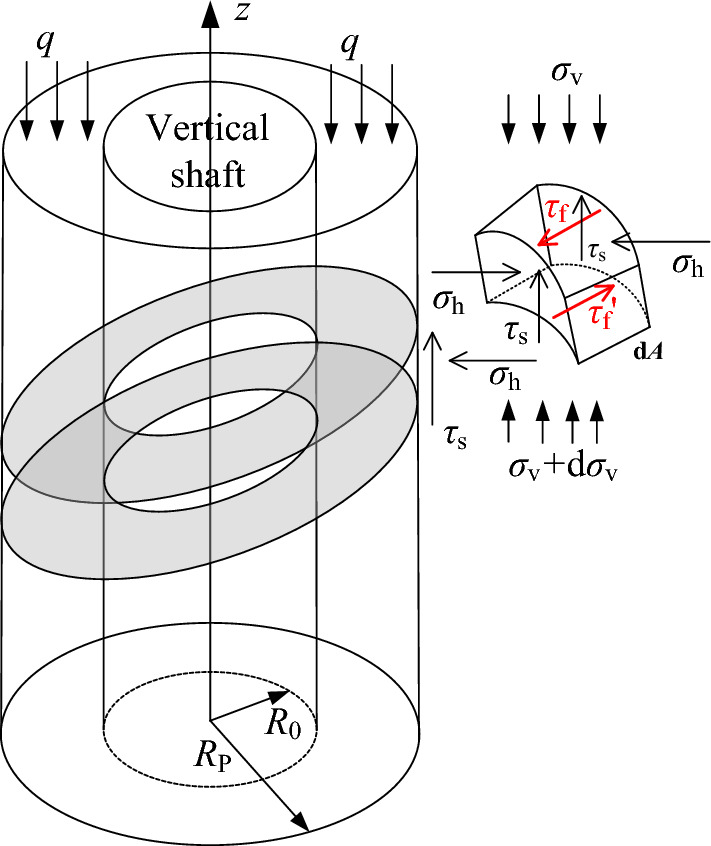
Calculation model of axisymmetric stratification.

The fracture circle radius ($R_{{\text{p}}}$) is still taken according to the Kastelner equation, and the unit body is taken as a unit radian. The calculation model is shown in Fig. 7.

The equilibrium differential equation of micro unit can be established as follows

15 $$\begin{gathered} \gamma (R_{{\text{p}}}^{2} - r_{{0}}^{2} ){\text{d}}z = - \frac{{1}}{\cos \theta }\sigma_{{\text{v}}} (R_{{\text{p}}}^{2} - r_{{0}}^{2} ) + \frac{{1}}{\cos \theta }(\sigma_{{\text{v}}} + {\text{d}}\sigma_{{\text{v}}} )(R_{{\text{p}}}^{2} - r_{{0}}^{2} ) + (R_{{\text{p}}} - r_{{0}} )\tau_{{\text{s}}} {\text{d}}z \hfill \\ \begin{array}{*{20}c} {} & {} & {} & {} & {} \\ \end{array} - \sigma_{{\text{v}}} (R_{{\text{p}}}^{2} - r_{{0}}^{2} )f\sin \theta + (\sigma_{{\text{v}}} + {\text{d}}\sigma_{{\text{v}}} )(R_{{\text{p}}}^{2} - r_{{0}}^{2} )f\sin \theta , \hfill \\ \end{gathered}$$

The boundary conditions are $z = 0,\sigma_{{\text{v}}} = q$ , and the surrounding rock pressure of the vertical shaft can be expressed as

16 $$\sigma_{{\text{h}}} = \left[ {K_{{\text{a}}}{\prime} q - \frac{{F(\gamma - \frac{C}{F})}}{\tan \varphi }} \right]{\text{e}}^{{ - z\frac{{K_{{\text{a}}}{\prime} \tan \varphi }}{D} \times k_{{\text{f}}} }} + \frac{{F(\gamma - \frac{C}{F})}}{\tan \varphi },$$

where, $F = \frac{{R_{p}^{2} - r_{{_{0} }}^{2} }}{{2(R_{p}^{{}} + r_{{_{0} }}^{{}} )}}$, and when $f = 0,\theta = 0$, Eq. (16) degenerates to Eq. (4).

### Calculation of surrounding rock pressure of vertical shaft considering inclined rock strata joints

Rock mass is composed of rock blocks and joints. The mechanical properties of joints are much lower than those of rock blocks, which control the strength of rock mass. The micro unit is replaced with a single layer of layered rock mass. The analysis method of Eq. (16) is used to derive the surrounding rock pressure of the vertical shaft, considering the inclined rock strata joints. Equation (16) can be regarded as an extension of the axisymmetric layering method. The cohesion of the joint is $C_{{\text{j}}}$, and the internal friction angle is $\varphi_{{\text{j}}}$, The radius of the fracture circle is still $R_{{{\text{p}}i}}$ according to the Kastelner equation, and the surrounding rock pressure can be expressed as

17 $$\sigma_{{\text{h}}} = \left[ {K_{{\text{a}}}{\prime} q - \frac{{F(\gamma - \frac{{C_{{\text{j}}} }}{F})}}{{\tan \varphi_{{\text{j}}} }}} \right]{\text{e}}^{{ - z\frac{{K_{{\text{a}}}{\prime} \tan \varphi_{{\text{j}}} }}{D} \times k_{{\text{f}}} }} + \frac{{F(\gamma - \frac{{C_{{\text{j}}} }}{F})}}{{\tan \varphi_{{\text{j}}} }},$$

### Engineering example analysis

**Figure 8:**
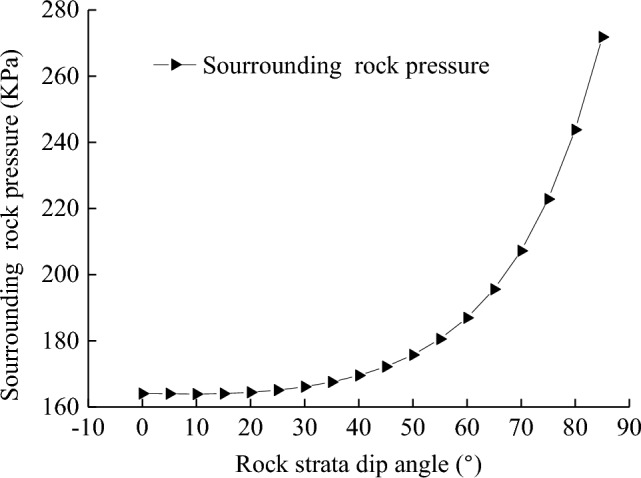
Calculation example of surrounding rock pressure of vertical shaft with different rock inclination ($z$ = 10m).

Taking Balasu mine in Inner Mongolia China as an example, the calculation results are obtained as shown in Fig. 8.

1. Figure 8 shows that the influence of the dip angle of the rock strata on the surrounding rock pressure is obvious. When the dip angle increases from 0° to 45°, the surrounding rock pressure increases slowly, and when the dip angle increases from 45° to 75°, the surrounding rock pressure increases rapidly.
2. When the shaft depth is 50 m, the exponential term of Eq. (17) is near 0, and the influence of the rock strata dip angle on the surrounding rock pressure does not conform to engineering practice, so Eq. (17) is no longer applicable.

## Conclusion

1. The calculation results indicate that the surrounding rock pressure of vertical shaft using the loose medium theory is far greater than the measured value, which is unsuitable for the calculation of the surrounding rock pressure in the bedrock section. The heavy liquid and Qin’s formula are suitable for shallow topsoil, and cylindrical retaining and sandwich wall theories are suitable for deep topsoil with low cohesion.
2. The axisymmetric layering method is suitable for bedrock strata, and its calculated results are near the measured values. The engineering measured data show that the influence of the rock strata buried depth on the surrounding rock pressure is not as significant as that of lithology, but the general trend shows an increase with the depth, which also verifies the applicability of the axisymmetric stratification method.
3. The axisymmetric layering method is further extended. Using Terzaghi theory’s idea of stress transfer, the calculation formula of the surrounding rock pressure of the vertical shaft in the inclined rock strata with single group joints is derived. This formula can reflect the influence of the dip angle and supplement the calculation of the surrounding rock pressure of the vertical shaft in the shallow rock strata.

## Data Availability

The data used to support the findings of this study author upon request. If someone would like to receive data from this study, please contact Yuanwu Sun via this email 2567887034@qq.com.
